# Supplementary figures and images for: Novel trajectory clustering method based on distance dependent Chinese restaurant process
Source: PeerJ Comput Sci. 2019 Aug 12;5:e206. doi: 10.7717/peerj-cs.206 (PMC7924552; doi:10.7717/peerj-cs.206)

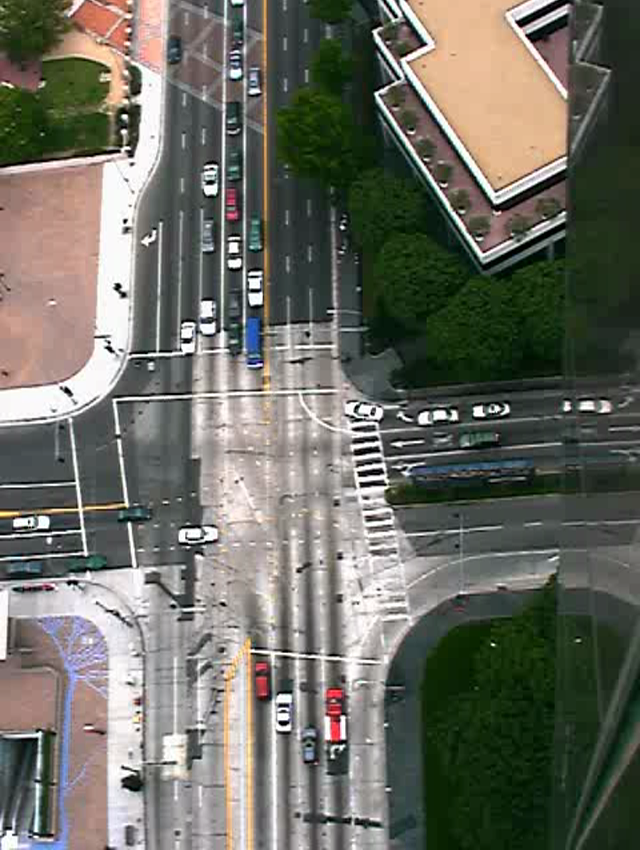

Supplement: Supplemental Information 1 — Programming code [file peerj-cs-05-206-s001.zip › data/Lankershim/ngsim.png]
