# Supplementary material for: Novel trajectory clustering method based on distance dependent Chinese restaurant process
Source: PeerJ Comput Sci. 2019 Aug 12;5:e206. doi: 10.7717/peerj-cs.206 (PMC7924552; doi:10.7717/peerj-cs.206)
Supplement: Supplemental Information 2 — Additional information about Equation 6. [file peerj-cs-05-206-s002.docx]

Novel trajectory clustering method based on distance dependent Chinese restaurant process

Reza Arfa^1, 2^, Rubiyah Yusof^1, 2^, Parvaneh Shabanzadeh^1, 2^

^1^ Centre for Artificial Intelligence and Robotics, Universiti Teknologi Malaysia, 54100 Kuala Lumpur, Malaysia

^2^ Centre for Artificial Intelligence and Robotics, Malaysia-Japan International Institute of Technology (MJIIT), Universiti Teknologi Malaysia, 54100, Kuala Lumpur, Malaysia

Corresponding Author:

Rubiyah Yusof^1, 2^, Reza Arfa^1, 2^

Email address: rubiyah.kl@utm.my, rezaarfa@gmail.com.

**Supplementary Material**

This supplementary material provides more detail about collapsed Gibbs sampling procedure given in Equation 6.

The steps involved in resampling $c_{i}$ are removing $c_{i}$’s outgoing link and resample its new outgoing link using Equation 6. There are only two scenarios that might happen when resampling $c_{i}$. The first one happens when the new outgoing link does not change the customer partition. This scenario happens if $c_{i}$ links to itself (i.e. $c_{i}=i$) or other customers belonging to the same partition of$c_{i}$. In this case, the partition remains the same. Therefore, no further action is required. Another possible scenario is when a new outgoing link joins two partitions. Since in this scenario the customer partition has changed, the values of prior and likelihood of partitions will also change and need to get updated.

By substituting Equations 2 and 5 to Equation 6 the collapsed sampling to resample $c_{i}$ can be compactly represent as follow:

$P(c_{i}=j|c_{-i}, \boldsymbol{X}_{1:N}, \boldsymbol{D}, f, \tau, \gamma,\gamma_{0})\propto\left\{ \begin{aligned} \tau, if c_{i}=i \\ f\left( d_{ij} \right), if c_{i}=j doesnot join tables \\ f\left( d_{ij} \right)\Omega\left( \boldsymbol{X}, z\left( c_{-i} \right)|G_{0} \right), if c_{i}=j joins tables k and l \end{aligned} \right.$ (1A)

Where

$\Omega\left( \boldsymbol{X}, z\left( c_{-i} \right)|G_{0} \right)= \frac{P(\boldsymbol{X}_{z^{k}(c_{-i})\cup z^{l}(c_{-i})}|G_{0})}{P(\boldsymbol{X}_{z^{k}(c_{-i})}|G_{0})P(\boldsymbol{X}_{z^{l}(c_{-i})}|G_{0})}$ (2A)

Using the Dirichlet-Multinomial Conjugacy, $P\left( \boldsymbol{X}_{z^{k'}\left( c_{-i} \right)} | G_{0} \right)$ can be written as (Teh, Newman, and Welling 2007)

$P\left( \boldsymbol{X}_{z^{k'}\left( c_{-i} \right)} | G_{0} \right)= \int P\left( \boldsymbol{X}_{z^{k'}\left( c_{-i} \right)} | \varphi_{k} \right)P\left( \varphi_{k} | G_{0} \right)d\varphi_{k'}= \frac{\Gamma(M\alpha_{0})\prod_{m} \Gamma(n_{k'}^{m}+\alpha_{0})}{\Gamma(n_{k}+M\alpha_{0})\Gamma^{M}(\alpha_{0})}$ (3A)

Where in this equation $\Gamma(.)$ is the gamma function, $\alpha_{0}$ is the concentration parameter of symmetric Dirichlet distribution, $M$ is the total number of grid cells, $n_{k}^{m}$ is the number of times $m$th grid cell is observed in the table $k$, and $n_{k}$ is the number of all grid cells assigned to table $k$.
